# Prostatic sarcoma after treatment of rectal cancer

**DOI:** 10.1186/1477-7819-5-82

**Published:** 2007-07-30

**Authors:** Salah M Abbas, Andrew G Hill

**Affiliations:** 1 Department of Surgery, Middlemore Hospital, University of Auckland, New Zealand

## Abstract

**Background:**

The relationship between radiation exposure for treatment of cancer and occurrence of a second primary cancer at the irradiated site is well known. This phenomenon is however rare in prostate.

**Case presentation:**

A 75-year-old farmer was treated for rectal cancer with preoperative 45 Gy of radiotherapy and abdominoperineal resection. Four years later he developed symptoms of bladder outlet obstruction and acute urinary retention. He underwent a transurethral resection of the prostate. Histological examination of the removed prostate tissue and immunohistochemistry revealed it to be a poorly differentiated sarcoma.

**Conclusion:**

We believe this to be the first reported case of radiation-induced sarcoma following radiotherapy treatment for rectal cancer. Since radiotherapy plays a pivotal role in the contemporary treatment of rectal adenocarcinoma, it is relevant to be aware of the potential long-term carcinogenic complications of radiotherapy of the pelvis.

## Background

Many cancer patients will receive radiation in the course of their cancer treatment [1]. With this increased use of adjuvant radiation, radiation induced cancer has become a well-recognized phenomenon. The relationship between radiation exposure and excess cancer incidence has been well documented for a wide variety of carcinomas and sarcomas in various sites.

Soft tissue sarcoma is a well-recognized complication following radiation exposure. The risk is thought to be 1% of patients [2]. Current modalities target tumour precisely but actually increase the overall amount of normal tissue exposed to low to moderate-dose ionising radiation [3]. As increasing numbers of patients with early-stage cancer are treated with radiation and survive longer, it seems likely that incidence of radiotherapy (RT)-induced soft tissue sarcoma will increase [4].

There is a latency period between radiation and the occurrence of soft tissue sarcoma. The cut-off is thought to be at least four years following radiation treatment. This latency period is necessary to differentiate a RT-induced sarcoma from a second primary that may predate the radiation treatment because no accurate molecular or pathologic markers exist. Thus any spontaneous sarcoma that appears in a radiation field is considered to be a radiation-induced malignancy [5]. Current estimates suggest that RT-induced sarcomas account for between 2.5 to 5.5% of all sarcomas [5,6].

Pelvic radiation for cancers other than rectal cancer has been implicated in the development of variety of soft tissue tumours such as angiosarcoma of the small bowel [7], osteogenic sarcoma and soft tissue sarcomas [8] such as prostatic carcinosarcoma [9], leiomyosarcoma of the rectum [10], and uterine sarcoma [11]. In this case report we present what we believe to be the first case of prostate sarcoma following preoperative neoadjuvant radiation for cancer of the rectum.

## Case presentation

A previously healthy 75-year-old farmer presented with a two-month history of rectal bleeding. He also gave a history of mild tenesmus and a feeling of faecal urgency; he had been otherwise well. Rigid sigmoidoscopy revealed a rectal tumour at 10 cm from the anal verge situated on the anterior wall of the rectum. Biopsy of the lesion showed a moderately differentiated adenocarcinoma. Colonoscopy showed no further lesions in the colon. A magnetic resonance imaging (MRI) of the rectum showed that the tumour was extensive and abutted the fascia propria on the back of the prostate. It also appeared to involve the levator ani muscles. A staging CT scan showed no evidence of liver or lung metastases. He underwent preoperative radiotherapy in the form of fractionated doses of 45 Gy given five days a week for five weeks.

Six weeks after radiotherapy he underwent an ultra low Hartmann's resection (Abdomino-perineal resection) with a permanent colostomy. Pathological examination of the removed rectum showed a T3 tumour with no lymph node involvement. He recovered well from surgery and managed his colostomy with no difficulty. Regular clinic follow-up was conducted and he continued to be well.

Four years later he developed symptoms of bladder outlet obstruction and acute urinary retention. He underwent a transurethral resection of the prostate. Histological examination of the removed prostate tissue showed prostate tissue and an anaplastic malignant tumour extensively infiltrating over 50% of the prostate tissue. Prominent within the tumour were large multinucleated cells with frequent, atypical, mitotic figures with extensive coagulative necrosis.

There was no evidence of gland formation within the tumour and specific features of colorectal carcinoma were not identified. Immunostaining showed the tumour was negative for epithelial markers cytokeratin 7, cytokeratin 20, cytokeratin 34B12, CEA and PSA. The tumour was also negative for the melanoma marker S100, the lymphocyte marker LCA, the muscle marker MSA, and the syncytiotrophoblast marker Beta HCG. The histological and immunohistochemical features were therefore those of a poorly differentiated sarcoma.

**Figure 1:**
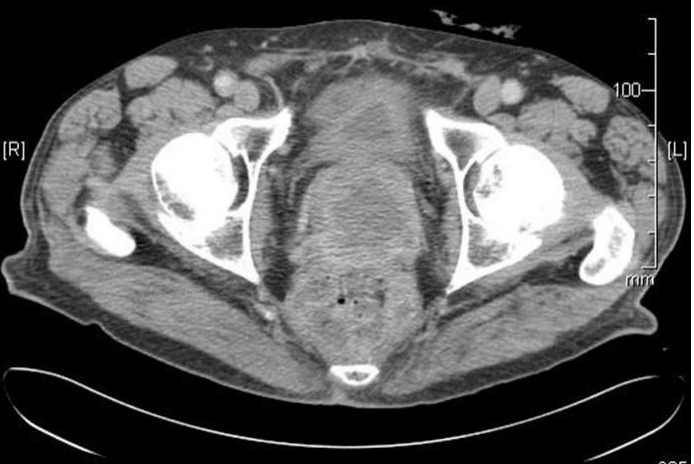
CT Scan of the Pelvis showing prostatic mass.

After the confirmation of sarcoma on tissue examination CT scan of the abdomen was performed. The scan (Figure 1) showed an enlarged and heterogenous prostate, with central fluid density. There was no local or distant adenopathy.

## Discussion

Rectal cancer is commonly treated with combination of surgical resection and radiotherapy, which is administered either before or after surgery [11,12]. The occurrence of prostatic sarcoma has not been reported before.

The diagnosis of post radiation sarcoma (PRS) is generally based on the following criteria:

• The histological features of the original lesion and PRS are completely different.

• PRS is located within the field of irradiation.

• Patients with cancer syndromes such as Li-Fraumeni and Rothmund-Thomson are excluded.

• The latent period (period between initiation of radiotherapy and histological diagnosis of second neoplasm) is more than 4 years. Although arbitrary given the wide age range reported in the literature (4–55 y), a period of 4 years generally has been accepted as being the lower limit for the latent period.

Medline was searched between 1966–2007 for rectal cancer, radiation therapy, and sarcoma, all as Medical Subject Headings. The relevant articles were reviewed for risk of carcinogenesis, treatment and prognosis of radiation-induced sarcoma following were retrieved. Marmion *et al*., have reported the development of a uterine carcinosarcoma more than 9 years after preoperative radiotherapy for rectal carcinoma [11]. Saggia *et al*., [13] reported a case of giant cell malignant fibrous histiocytoma in the pelvis 9 years following radiotherapy for rectal cancer. On the other hand a case of rectal adenocarcinoma has been described in a 9-year-old child following radiation treatment of pelvic rhabdomyosarcoma [14].

Radiation-induced sarcomas are thought to be aggressive, high-grade tumours, often with a poor prognosis. In a large series from the Memorial Sloan-Kettering cancer centre 90% of patients were able to undergo resection for curative intent; however, 46% of patients had either a gross or microscopically positive resection margin despite the fact that the majority of these tumours were less than 5 cm in diameter [5]. From the same group 80% of patients who had tumour resection had high-grade tumours, resulting in a 5-year survival of 34% for this subgroup of patients. Radiation induced sarcomas tend to be diffuse and infiltrate along tissue planes that have been made indistinct by the effect of previous radiation. Positive margins result in a 50% reduction in survival. This suggests aggressive and wide excision to achieve complete curative resection is required to improve the chances of long survival. Survival in radiation-induced sarcoma is generally worse than other sarcomas [15-18].

Radiation-induced sarcoma remains uncommon and arises in 0.035 to 0.2% of all irradiated patients [19]. The most common histological subtypes of radiation-induced sarcomas are osteogenic, malignant fibrous histiocytoma, angio- and lymphangiosarcoma and spindle cell sarcoma [19,20]. Despite the low incidence of radiation-induced sarcoma, it is expected to be seen more frequently, due to an increased life expectancy with progressively improved survival in cancer patients as a result of increased effectiveness of cancer therapy and better treatment regimes; however the dose-effect relationship between radiation and sarcoma is controversial [19-23]. Neither a minimum of cumulative radiation dose nor a correlation between modality of radiation and the incidence of radiation-induced sarcomas is reported in the medical literature, but it is generally agreed that high radiation dose is needed for development of sarcoma [24]. A radiation dose-response relationship has been demonstrated for all sarcomas and, for the first time in humans, for soft tissue sarcomas.

Several mechanisms by which radiation may induce genetic changes leading to malignant transformation have been proposed, but the exact development of radiation-induced cancer is still unclear. The loss of heterozygosity in directly radiated cell nuclei activate mutational occurrences in tumour suppressor genes resulting in malignant degeneration. Furthermore, genetic mutations may also be induced by cytoplasmic irradiation and the release of cytokines. Therefore, important genetic effects can also be observed in cells that did not directly receive nuclear radiation [24].

## Conclusion

Thus long-term follow-up is important for patients who have been treated with radiotherapy. Any suspicious lesion should be thoroughly assessed with imaging and tissue sampling to identify the nature of the lesion. If the lesion proves to be sarcoma the treatment of choice for radiation-induced sarcomas is surgical resection with clear gross and microscopic margins. Chemotherapy for radiation-induced sarcoma is not proven to result in prolonged survival [5].

While we have not established a conclusive relationship between the sarcoma of the prostate and the previous radiotherapy the association between the two seems to be more than coincidental and we wait with interest further reports from other institutions.

## Competing interests

The author(s) declare that they have no competing interests.

## Authors' contributions

SMA: Did the literature search, prepared the draft manuscript

AGH: Conceived the idea and edited the manuscript

All authors approved the final version of the manuscript

## References

[B1] Mark RJ, Poen J, Tran LM, Fu YS, Selch MT, Parker RG (1994). Postirradiation sarcomas. A single-institution study and review of the literature. Cancer.

[B2] Pierce SM, Recht A, Lingos TI, Abner A, Vicini F, Silver B, Herzog A, Harris JR (1992). Long-term radiation complications following conservative surgery (CS) and radiation therapy (RT) in patients with early stage breast cancer. Int J Radiat Oncol Biol Phys.

[B3] Hall EJ, Wuu CS (2003). Radiation-induced second cancers: the impact of 3D-CRT and IMRT. Int J Radiat Oncol Biol Phys.

[B4] Taghian A, de Vathaire F, Terrier P, Le M, Auquier A, Mouriesse H, Grimaud E, Sarrazin D, Tubiana M (1991). Long-term risk of sarcoma following radiation treatment for breast cancer. Int J Radiat Oncol Biol Phys.

[B5] Cha C, Antonescu CR, Quan ML, Maru S, Brennan MF (2004). Long-term results with resection of radiation-induced soft tissue sarcomas. Ann Surg.

[B6] Huvos AG, Woodard HQ, Cahan WG, Higinbotham NL, Stewart FW, Butler A, Bretsky SS (1985). Postradiation osteogenic sarcoma of bone and soft tissues. A clinicopathologic study of 66 patients. Cancer.

[B7] Aitola P, Poutiainen A, Nordback I (1999). Small-bowel angiosarcoma after pelvic irradiation: a report of two cases. Int J Colorectal Dis.

[B8] Huvos AG, Woodard HQ, Cahan WG, Higinbotham NL, Stewart FW, Butler A, Bretsky SS (1985). Postradiation osteogenic sarcoma of bone and soft tissues. A clinicopathologic study of 66 patients. Cancer.

[B9] Tseng TY, Sevilla DW, Moul JW, Maloney KE (2006). Prostatic carcinosarcoma 15 years after combined external beam radiation and brachytherapy for prostatic adenocarcinoma: a case report. Prostate Cancer Prostatic Dis.

[B10] Caporale A, Angelico F, Cosenza MU, Giuliani A, Del Ben M, Benvenuto E, Franchi F (2003). A late complication of pelvic radiotherapy: leiomyosarcoma of the rectum. Report of a case and review of the literature. Hepatogastroenterology.

[B11] Marmion PJ, Goldfarb PM, Youngkin TP (1981). Uterine sarcoma following adjuvant radiotherapy for rectal carcinoma. J Surg Oncol.

[B12] Curigliano G, Spitaleri G, Zampino G, Eriksen MT, Wibe A, Haffner J, Wiig JN, on behalf of The Norwegian Rectal Cancer Group (2007). Prognostic groups in 1,676 patients with T3 rectal cancer treated without preoperative radiotherapy. Dis Colon Rectum.

[B13] Saggia C, Forti G, Biaggi G, Lattuada S, Santagostino A, Angeli G, Pollo MC, Negru ME, Alabiso O (2004). Two cases of secondary soft tissue sarcomas after radiotherapy and radiochemotherapy. Tumori.

[B14] Kalteis T, Heers G, Elsner R (2005). Adenocarcinoma of the rectum in childhood following chemotherapy and radiotherapy for a rhabdomyosarcoma – a case report. Eur J Pediatr Surg.

[B15] Lagrange JL, Ramaioli A, Chateau MC, Marchal C, Resbeut M, Richaud P, Lagarde P, Rambert P, Tortechaux J, Seng SH, de la Fontan B, Reme-Saumon M, Bof J, Ghnassia JP, Coindre JM (2000). Sarcoma after radiation therapy: retrospective multiinstitutional study of 80 histologically confirmed cases. Radiation Therapist and Pathologist Groups of the Federation Nationale des Centres de Lutte Contre le Cancer. Radiology.

[B16] Singer S, Antonescu CR, Riedel E, Brennan MF (2003). Histologic subtype and margin of resection predict pattern of recurrence and survival for retroperitoneal liposarcoma. Ann Surg.

[B17] Koea JB, Leung D, Lewis JJ, Brennan MF (2003). Histopathologic type: an independent prognostic factor in primary soft tissue sarcoma of the extremity?. Ann Surg Oncol.

[B18] Brady MS, Gaynor JJ, Brennan MF (1992). Radiation-associated sarcoma of bone and soft tissue. Arch Surg.

[B19] Brenner DJ, Curtis RE, Hall EJ, Ron E (2000). Second malignancies in prostate carcinoma patients after radiotherapy compared with surgery. Cancer.

[B20] Sale KA, Wallace DI, Girod DA, Tsue TT (2004). Radiation-induced malignancy of the head and neck. Otolaryngol Head Neck Surg.

[B21] Demirkan F, Ünal S, Cenetoglu S, Cinel L (2003). Radiation-induced leiomyosarcomas as second primary tumours in the head and neck region: report of 2 cases. J Oral Maxillofac Surg.

[B22] Little JB (2000). Radiation carcinogenesis. Carcinogenesis.

[B23] König O, Bockmühl U, Lammert I (2001). Radiation-associated malignant fibroushistiocytoma of the oropharynx. Head Neck Oncol.

[B24] Inoue YZ, Frassica FJ, Sim FH, Unni KK, Petersen IA, McLeod RA (2000). Clinicopathologic features and treatment of postirradiation sarcoma of bone and soft tissue. J Surg Oncol.

[B25] Wong FL, Boice JD, Abramson DH, Tarone RE, Kleinerman RA, Stovall M, Goldman MB, Seddon JM, Tarbell N, Fraumeni JF, Li FP (1997). Cancer incidence after retinoblastoma. Radiation dose and sarcoma risk. JAMA.

